# Impact of Cryopreservation and Freeze-Thawing on Therapeutic Properties of Mesenchymal Stromal/Stem Cells and Other Common Cellular Therapeutics

**DOI:** 10.1007/s40778-022-00212-1

**Published:** 2022-04-27

**Authors:** Chasen Cottle, Amanda Paige Porter, Ariel Lipat, Caitlin Turner-Lyles, Jimmy Nguyen, Guido Moll, Raghavan Chinnadurai

**Affiliations:** 1 Department of Biomedical Sciences, Mercer University School of Medicine, Savannah, GA USA; 2 BIH Center for Regenerative Therapies (BCRT) and Berlin Brandenburg School of Regenerative Therapies (BSRT), Berlin Institute of Health (BIH), Charité Universitätsmedizin Berlin, corporate member of Freie Universität zu Berlin and Humboldt-Universität zu Berlin, Berlin, Germany

**Keywords:** Cellular therapeutics, Cryopreservation, Freeze-thawing, Safety and efficacy, Functionality, Mesenchymal Stromal/Stem Cells (MSCs), Stem cells (MSCs), Effector T cells (Teff), Regulatory T cells (Treg), Natural killer (NK) cells, Induced pluripotent stem cells (iPSCs)

## Abstract

***Purpose of Review*:**

Cryopreservation and its associated freezing and thawing procedures–short “freeze-thawing”–are among the final steps in economically viable manufacturing and clinical application of diverse cellular therapeutics. Translation from preclinical proof-of-concept studies to larger clinical trials has indicated that these processes may potentially present an Achilles heel to optimal cell product safety and particularly efficacy in clinical trials and routine use.

***Recent Findings*:**

We review the current state of the literature on how cryopreservation of cellular therapies has evolved and how the application of this technique to different cell types is interlinked with their ability to engraft and function upon transfer in vivo, in particular for hematopoietic stem and progenitor cells (HSPCs), their progeny, and therapeutic cell products derived thereof. We also discuss pros and cons how this may differ for non-hematopoietic mesenchymal stromal/stem cell (MSC) therapeutics. We present different avenues that may be crucial for cell therapy optimization, both, for hematopoietic (e.g., effector, regulatory, and chimeric antigen receptor (CAR)-modified T and NK cell based products) and for non-hematopoietic products, such as MSCs and induced pluripotent stem cells (iPSCs), to achieve optimal viability, recovery, effective cell dose, and functionality of the cryorecovered cells.

***Summary*:**

Targeted research into optimizing the cryopreservation and freeze-thawing routines and the adjunct manufacturing process design may provide crucial advantages to increase both the safety and efficacy of cellular therapeutics in clinical use and to enable effective market deployment strategies to become economically viable and sustainable medicines.

## Introduction

Cellular therapeutics are living medicines, which belong to an innovative new class of drugs called advanced therapy medicinal products (ATMPs, medicines for human use that are based on genes, cells or tissues) in Europe and furthermore frequently referred to as clinical cellular therapeutics (CCTs) in North America [1]. In this review article, we give an update on the impact of “cryopreservation” and “freeze-thawing” procedures on ATMPs/CCTs, with an emphasis on some of the most common cellular therapeutics in use today.

**Fig. 1:**
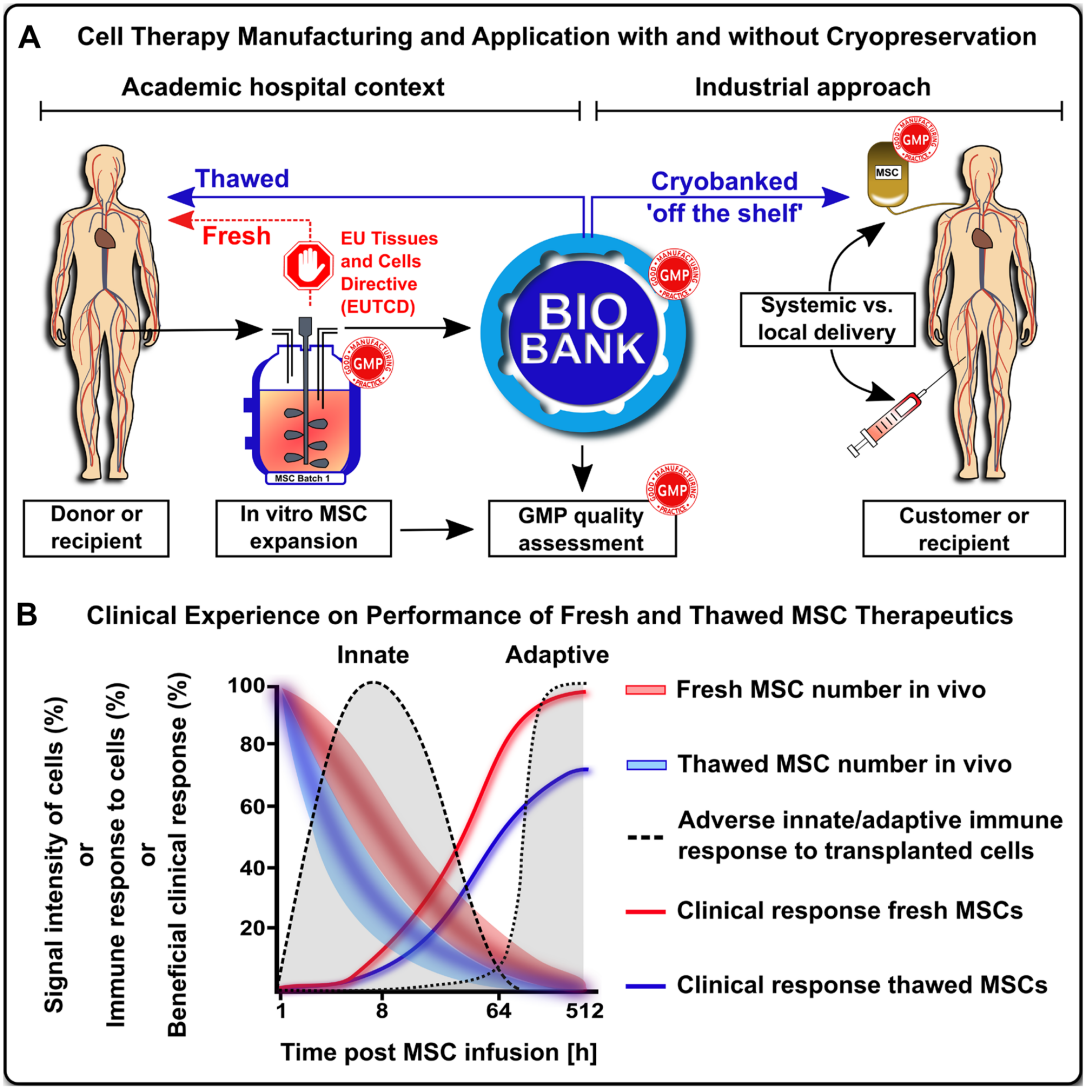
Impact of cryopreservation and freeze-thawing on clinical cell therapies. (**A**) Cell therapy manufacturing and application with and without cryopreservation: Cell-based advanced therapy medicinal products (ATMPs), such as mesenchymal stromal / stem cells (MSCs) are typically generated from human donor tissue by selective in vitro expansion under environmentally controlled conditions, e.g., by employing advanced bioreactors. The cell batches are then typically stored in a cryopreserved state and subjected to good-manufacturing-practice (GMP) quality controls, in order to obtain batch release for clinical use. The therapeutic cells are then delivered to patients most commonly either by local or systemic injection [3, 4]. Predominantly freeze-thawed cells delivered shortly post cryoretrieval were used in the past. The optional clinical use of fresh cells has been mainly suspended in Europe since 2007, due to the adoption of the European Union Tissues and Cells Directive (EUTCD) although conditional exemptions can be warranted, e.g., under use of the European Hospital Exemption [9•]; and (**B**) Clinical Experience and Performance of Fresh and Thawed MSC Therapeutics: Therapeutic cell engraftment, immune, and clinical response evaluation in animal models and patient cohorts suggest that the therapeutic activity of MSCs is not the result of long-term engraftment and tissue formation, but probably the result of a complex (immune)-modulatory action exerted within the first hours to days and weeks of infusion. Differences in transient engraftment / persistence (signal intensity of cells, %), different susceptibility to triggering of the instant blood-mediated inflammatory reaction (IBMIR) / innate and adaptive immune responses (adverse immune response, %), and different bioactivity and environmental responsiveness may provide an explanation, why fresh minimal expanded cells appear to have favorable therapeutic activity [12]

**Fig. 2:**
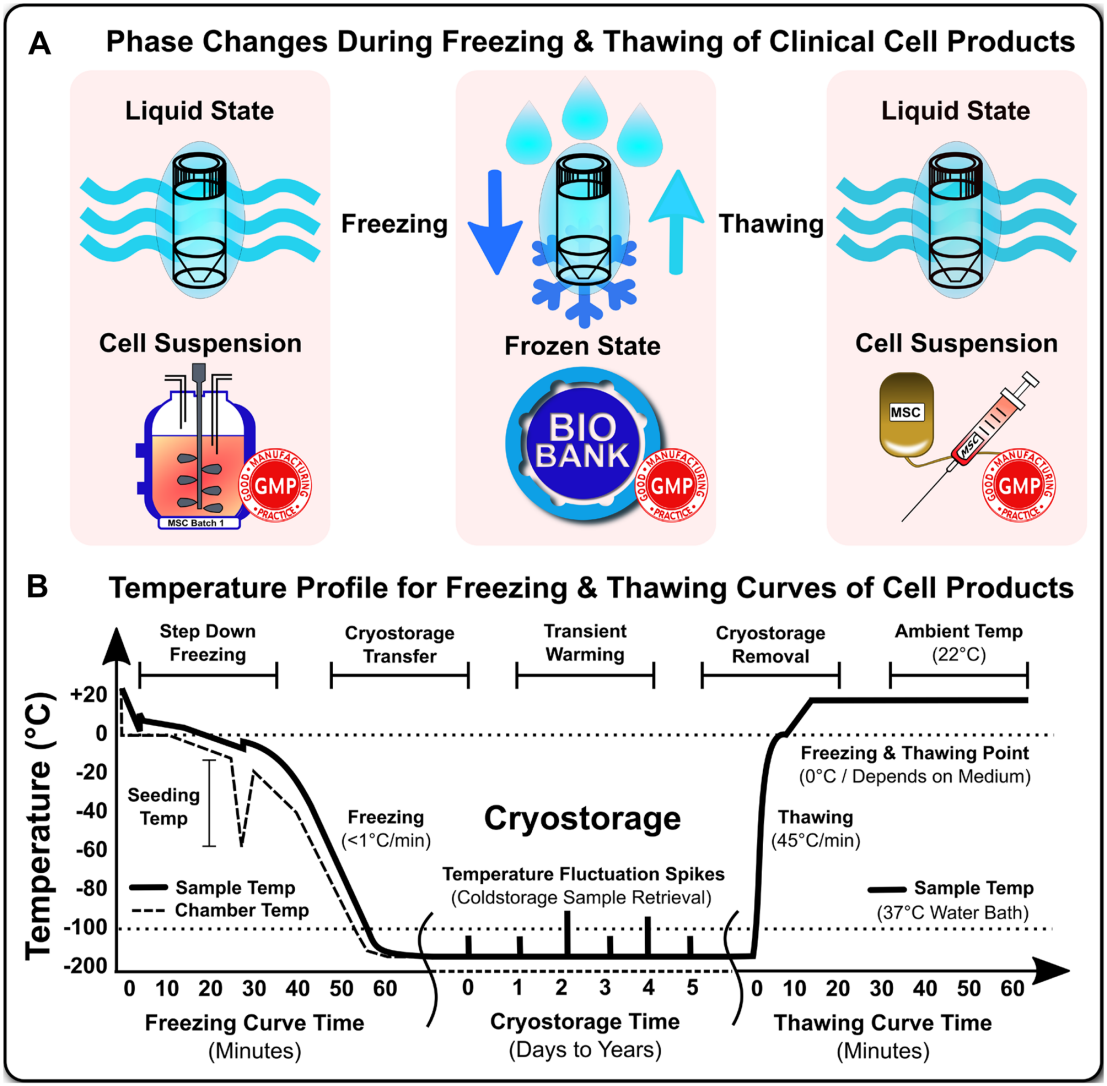
Cryopreservation and freeze-thawing of cell products. (**A**) Phase changes during freezing and thawing of clinical cell products: For permanent long-term cryostorage in a frozen state in a biobank the clinical cell product (e.g., mesenchymal stromal/stem cells; MSCs) has to be first harvested (e.g., from a suspension bioreactor) and then frozen under controlled conditions with the cells concentrated and resuspended in a suitable highly optimized cryomedia that prevents cell damage and supports robust thawing with amenable cell recovery, viability, and functionality for clinical use as an infusion or tissue injection product (cell infusion bag and cell injection syringe) with all steps being performed under good manufacturing practice (GMP) conditions; and (**B**) Temperature profile for freezing and thawing curves of cell products: A crucial aspects in cryopreservation of clinical cell products is the optimal temperature profile and speed of the freezing and thawing curve (e.g., freezing of MSCs is typically done in a controlled rate automated freezing device at < 1*C/min with appreciation of the seeding temperature to prevent harmful ice crystal formation and allowing for constant cooling, while thawing is typically done in 37*C water baths with optimal heat transfer at 45*C/min, which allows fairly rapid thawing to ambient temperature (e.g., room temperature 22*C). Once frozen, the cell product can be transferred to ultra low temperature cryostorage (Less than -135 to -196*C) and maintained in an inert state for years. However, temperature fluctuation spikes due to frequent sample retrieval can also slowly degrade / damage frozen products

Cell-based therapeutics are typically isolated from the human body and then selectively expanded in a good manufacturing practice (GMP)-conform manufacturing facility. They are then commonly cryopreserved for long-term storage until clinical use and applied/delivered to the patient either as systemic infusion or injection (Figs. 1A–B and 2) [2–7], where they are intended to perform healing responses and several other therapeutic functions.

Due to the different modes of manufacturing and clinical delivery, there are several issues that differ between living drugs and conventional pharmaceuticals. A key factor is the need for ‘living’ cell storage, e.g., “cryopreservation” and the adjunct “freezing and thawing” procedures of living cells for clinical use [8]. The terminology and characteristics of these processes, which may differentially impact on therapeutic cell function and mechanism of action (MoA), are explained in more detail in the “Key Terminology in “Cryopreservation” and “Freeze- Thawing” of Cell Products” section (Fig. 2 and Table 1) [2, 9•, 10–13, 14•, 15, 16, 17••, 18–22].

**Table 1:** Comparison of fresh and frozen MSCs in vitro and in vivo. Adapted from [12]

**Cellular properties**	**Comparison fresh and frozen cells shortly post cryorecovery**
**Cell growth and cell differentiation in vitro**	Most studies reported no difference for in vitro cell growth and differentiation between fresh or freeze-thawed cells
**Phenotype, viability, bioactivity in vitro, and bioactivity in vivo**	Majority of studies reported no differences, but a substantial number of studies also reported differences, which can at least in part be restored by cryorecovery or preconditioning strategies
**Engraftment in vivo** **Biodistribution in vivo** **Adverse events in vivo**	Very limited information published for each of the categories. Two studies found differences, which could also be restored by cryorecovery or preconditioning strategies

Some of the first principles for cellular therapy were established in line with the early progress made in hematopoietic stem cell transplantation (HSCT). HSCT was initially developed to counter the harmful effects of radiation on humans and for replacing lost or defective function of the hematopoietic system in treatment of blood cancer and many other harmful pathologies [23]. Principle discoveries that paved the way for both HSCT and cellular therapies entail several Nobel-Prize winning concepts in daily use today, such as the human ABO blood group system (Nobel Prize in Physiology and Medicine awarded to Karl Landsteiner in 1930) and the human leukocyte antigen (HLA) system (Nobel Prize in Physiology and Medicine awarded to E. Donnall Thomas and Joseph E. Murray in 1990) [11, 23], which are now part of the foundation for modern transfusion medicine, tissue typing and transplantation [24].

**Fig. 3:**
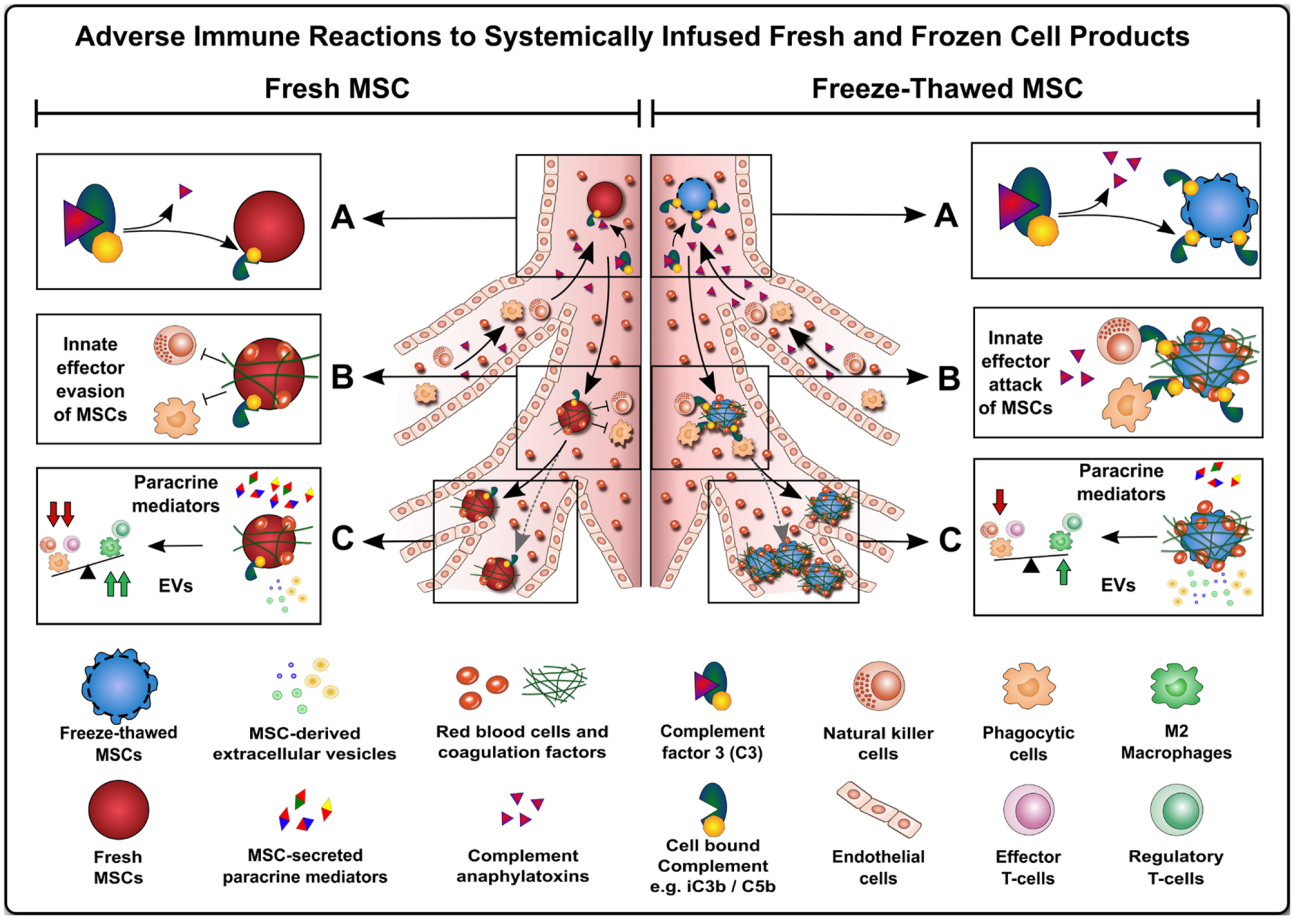
Adverse immune reactions to systemically infused fresh and frozen cell products. Studies on the biodistribution and time course of MSC engraftment upon clinical delivery via systemic intravenous infusion to patients suggest that most cells are rapidly embolized within the lung microvasculature and destroyed shortly post infusion due to a number of antagonizing mechanisms, e.g., triggering of innate immune cascades and the associated thromboinflammation including activation of innate and adaptive effector cells, a sequence of events commonly summarized under the term “Instant Blood-Mediated Inflammatory Reaction” (IBMIR) [3, 9•, 79, 145]. (**A**) Instant Immune Recognition of Infused Fresh vs. Freeze-Thawed MSCs: While freshly harvested cells display optimal cell membrane physiology, freeze-thawed cells readily derived from cryostorage can display a disturbed cell membrane physiology, depending on the specifics of the cryopreservation and cryorecovery procedures used at site. This makes freeze-thawed cells more prone to hemodynamic disruption and innate immune attack, here exemplified through complement activation and coating with sequential complement component 3 (C3)-activation products (e.g., C3b, iC3b, C3d) and the concomitant release of complement C3 and C5 anaphylatoxins (e.g., C3a and C5a) [9•, 10, 11]. (**B**) Innate Effector Cell Engagement by Fresh and Freeze-Thawed MSCs: The complement opsonins and anaphylatoxins are potent activating ligands for innate effector cells, such as phagocytes and NK-cells, which can attack and damage therapeutic cells, e.g., through release of perforin and granzyme, leading to triggering of target cell apoptosis, membrane lysis, and cell death. Furthermore, depending on the initial tissue source MSCs display varying levels of procoagulant tissue factor (TF/CD142) [3, 146], which is a potent trigger of the coagulation cascade, and can thus promote embolization and sequestration of cellular therapeutics in the microvasculature, which is less evident for fresh cells. (**C**) Differential Release or Paracrine Mediators and Microparticles: Differential susceptibility of fresh and thawed MSCs to innate immune attack promotes their differential release of 1) Paracrine mediators (e.g., cytokines, chemokines, and small sized metabolites) and 2) MSC-derived microparticles (e.g., exosomes 70-150 nm, microvesicles 100 nm to 1um, and apoptotic bodies 1-5um) [147, 148]. The stronger innate attack and faster killing of freeze-thawed MSCs limits their active secretion of soluble paracrine mediators but augments their passive release of cell-derived microparticles. In turn, fresh MSCs exhibit longer in vivo persistence and paracrine secretion, but less microparticle secretion. Preclinical and clinical data suggest that an active response by metabolically active fresh cells elicits stronger beneficial immune modulation

The original concept of HSCT and cellular therapies was motivated by the assumption that the infused cells need to engraft short or long-term to exhibit full functionality in the human body to execute their healing and regenerative properties [25–27]. While, long-term engraftment and reconstitution of the hematopoietic system is crucial for HSCT long-term function, often only a transient in vivo engraftment is achieved with many other types of cellular therapeutics. This applies particularly for the suboptimal survival/long-term engraftment of some types of freeze-thawed cells, e.g., mesenchymal stromal / stem cells (MSCs) (Figs. 1B and 3) [9•, 12, 28]. This may also apply to some degree for effector and regulatory T-cell therapeutics (Teffs and Tregs) and chimeric antigen receptor (CAR) augmented T cell and natural killer (NK) cell products (CAR-T and CAR-NK cell products, respectively) [13, 14•, 15, 16, 29, 30].

Intriguingly, one of the first lessons learnt from HSCT: That long-term-engraftment is beneficial, and the generalized paradigm derived therefrom: That engraftment is desired for all types of cell therapy, may not always hold true. Indeed, we may now witness a paradigm shift: That apoptosis is part of MSCs MoA [2, 9•, 12, 31, 32]. This may indicate that historical lessons / clinical experience cannot always be transferred 1:1 when employing different cell types, as also observed in other settings [2, 3, 5–7, 33]. Desirable properties and translational variables (e.g., product characterization parameters, time and mode of delivery, cryopreservation protocols, monitoring of the clinical response, and prediction of therapeutic success etc.) may have to be defined individually for each therapeutic cell product type [2, 4, 34], as done in this review.

Thus, the actual need for therapeutic cell engraftment in vivo and their consequent cellular function, based on their metabolic activity and the active responsiveness of living cells (which is characteristically interlinked with their selective cell-intrinsic properties), is in part contested today for some novel therapeutics, such as MSCs [35–37]. In contrast to HSCT, long-term engraftment of MSCs may not always be required and at least in one therapeutic modality may be the result of a host response to apoptotic MSCs [2, 31, 32].

Recent findings also suggest that the immunomodulation and stimulation of regenerative healing responses could be an ancient physiological response of the mammalian body to apoptotic cells introduced in the blood stream (e.g., placenta-derived stromal cell shedding into the blood stream and embolization in the lungs), which may lead to a transient polarization the host immune system (e.g., alveolar macrophages) to execute the beneficial responses observed upon MSC application [35, 38]. Thus, each cell type has its distinct intrinsic properties / potential to elicit host modulation and translation variables that must be considered in cell therapy.

Irrespective of the need for therapeutic cell engraftment, current manufacturing strategies still mainly attempt to produce cellular therapeutics with preferential engraftment properties in their aim to develop potent and effective cellular pharmaceuticals [12, 29]. Cryopreservation is a widely used strategy at the cell manufacturing facilities where therapeutic cells are cryobanked in liquid nitrogen tanks until needed for infusion into patients.

This is a feasible approach from an economical and regulatory standpoint, since release criteria of therapeutic cells can be determined well in advance, and can be used as an “off-the-shelf” product [14•]. Economical and regulatory feasibility are strong arguments for the use of frozen “off-the-shelf” products, if the clinical outcome is satisfactory [39]. They can then be shipped in frozen state to healthcare centers upon immediate need, where they can be thawed near bedside and infused into patients within a few hours post-thaw. Upon thawing, it is expected that the cells rapidly recover from their cryo-stunned phase to a functional state to perform healing functions.

Despite the apparent practical and economic feasibility of this approach, some concerns have emerged regarding the actual functionality/potency of the cryopreserved therapeutic cells that are infused immediately post-thaw into patients. Cells are the living units of tissues and organs within the human body where physiological integrity is maintained by environmental niches and cues, e.g., temperature, osmolarity, nutrients etc. [40, 41]. However, these cues necessary for cell function are or can be compromised during cryopreservation and this may compromise optimal cell function immediately or shortly post thawing [9•, 12, 14•].

In HSCT, the cell graft inherent hematopoietic stem and progenitor cells (HSPCs) are often thawed and infused into the patients to repopulate the entire hematopoietic system [42]. While the HSCT approach is proven successful, it should be noted that HSPCs have extensive self-renewing properties and even a small number of “stem cells” can multiply and accomplish the desired hematopoietic reconstitution [25]. In contrast, MSCs differ substantially from HSPCs, since they appear to execute therapeutic properties mainly as “hit and run” mechanism without lifelong persistence or engraftment [34]. The cells are mainly thought to exert their therapeutic function by instructing host cells through paracrine signals, thereby initiating a healing cascade or by modulating the balance between inflammation and anti-inflammation [41].

Thus, the impact of “cryopreservation” and “freeze-thawing” on the functionality of therapeutic cells varies substantially. In the following sections, we will give an update on its impact for the functionality of both hematopoietic and non-hematopoietic cellular therapeutics.

## Key Terminology in “Cryopreservation” and “Freeze-Thawing” of Cell Products

As illustrated in Fig. 2, the signature terms “cryopreservation” and “freeze-thawing” have distinct meanings and are not truly interchangeable [12]. The first term “cryopreservation” mainly relates to the whole process from the initial “freezing,” followed by “cold-storage” for a flexible amount of time, and eventually “thawing,” e.g., for clinical use of the cell product. Importantly, this includes the cold-storage period, e.g., long-term storage of the frozen cell product in liquid or vapor-phase nitrogen tanks/devices, which typically operate at -196 degrees Celsius or at -135 to -190 degrees, respectively, with minor differences in temperature stability. Alternatively, there also exists short- and long-term storage, e.g., in -80 and -180 degrees Celsius freezers and even colder ultralow temperature (ULT) freezers, respectively.

Cryopreservation cold-storage is often done for an uncertain period of time (e.g., days, months, or even years) with its associated artifacts that may be introduced during this time [8]. Below -135 degrees Celsius cell activity effectively stops, but transient warming events (e.g., due to frequent cell batch removal/introduction from the storage device) can potentially affect and even damage any cells remaining long-term in the cold-storage environment, in particular when the temperature is repeatedly raised to a point where cell quality may be affected/compromised. Such artifacts have to be anticipated during clinical use of frozen cell products in patients, in particular in smaller scale facilities. Thus, when using the term cryopreservation, the emphasis is more on the cold-storage period and its associated artifacts introduced during this period.

In turn, the terms “freezing” and “thawing” mainly refer to the phase changes from liquid to frozen state and vice versa, and here the emphasis is on the actual phase-change, in particular the ice nucleating point where exothermic reactions occur and when ice crystals form that can puncture and thus damage the cell membrane. These processes are associated with potential changes in osmolarity, pH, and temperature that have to be effectively controlled [43, 44]. The transient heating resulting from the exothermic reaction can damage the cells by denaturizing proteins and other cellular structures. Modern optimized cryopreservation media and freezing-devices minimize these detrimental effects (e.g., through optimal freezing-curves) [45].

Hence, the terms “cryopreservation” and “freeze-thawing” are associated with a minutely different emphasis on different process and their associated characteristics and specific problems. Indeed, the first term “cryopreservation” mainly focuses on consistent cold-storage in frozen state at a defined cold temperature, and the second term “freeze-thawing” focuses on the phase changes between liquid and frozen phase and how to reach or leave this state.

Although there is some redundancy between these two terms, they are not interchangeable. The understanding of the meaning of these two terms is very important for understanding the respective problems related to the different terminology, and we have furthermore illustrated the different key points highlighted in this paragraph in Fig. 2.

## Hematopoietic Stem and Progenitor Cells (HSPCs)

HSCT is a widely employed clinical practice with > 50.000 procedures conducted annually around the globe [46–48]. Bone marrow (BM), cord blood (CB), and peripheral blood (PB) are the most common sources for HSPCs used in HSCT [49]. Although HSPC grafts can be used fresh, modern routines often rely on cryobanking of BM, CB and PB grafts [50–54]. Particularly, CB and PB grafts are often stored cryobanked until clinical use [51, 55–57]. Experience during the COVID-19 pandemic suggests that even in the well-established HSCT setting, cryopreservation and increased transit times can detrimentally affect immune reconstitution [57, 58].

Alotaibi and coworkers have investigated the effect of cryopreservation of grafts on the clinical benefit of related and unrelated and haploidentical allogeneic HSCT [59]. No difference was identified with neutrophil engraftment, platelet engraftment, graft failure and grade II-IV acute GvHD. In addition, they reported that disease relapse was less in patients with mild or no chronic GvHD when fresh grafts were used. Another study showed a trend towards extensive chronic GvHD and delayed platelet engraftment with cryopreserved PBSC grafts from both related and unrelated donors [60]. The apparent contradictions in these two exemplary studies illustrate that further assessments are necessary to identify the true impact of fresh and cryo grafts on the clinical short- and long-term benefit post HSCT.

Cryopreservation and freeze-thawing procedures for HSPC grafts can vary from one cell processing facility to another according to regional preferences and no universal methodology is in practice to date [54]. Lecchi and Hornberger et al. discussed in great detail major variables that may affect function of HSPCs post thawing, which includes cryoprotectants, cell and cryoprotectant concentration, sample volume, freezing procedure, cooling rate, cell storage and thawing [42, 54]. Standard practices include the use of cryoprotectants (usually 3.5–10% dimethyl sulphoxide; DMSO) and in-house developed controlled freezing-rate procedures, and thawing either at the bedside or in the laboratory, to wash and remove cryoprotectants [54].

It has been suggested that cryopreserved HSPCs should be infused as soon as possible post thawing including the cryomedium, to preserve their optimal cell viability and function [42], but there is also literature recommending prior washing/removal of cryoprotectants, especially when patients are less than 20 kg of body weight [61].

Although DMSO-removal may be ideal to prevent adverse toxicity, the DMSO-washout risks cell graft contamination/damage and requires practical cell handling (time) at site. These factors increase the potential of inducing cellular apoptosis in the cell graft, which may delay the HSPC engraftment in patients [54].

Both, decreasing the percentage of DMSO during cryopreservation and diluting DMSO prior to infusion, are alternative methods to conventional washing [54], which may also be beneficial for fragile cellular therapeutics [14•]. In addition, novel methods of DMSO removal have also been employed / tested in some facilities, which includes automated commercial washing devices (e.g., Sepax^™^ device) and filtration spinning membranes, microfluidic channels, and hollow-fiber membranes that allow for DMSO removal without centrifugation [62].

A main concern with cryopreservation of HSPCs is decreased viability due to cell injury during freezing and thawing [54]. Such injuries can result from osmolarity disturbances, ice crystal formation, and cell dehydration [63]. Desoutter et al. demonstrated that cryopreservation decreases HSPC viability and mechanistic investigation identified that the caspase-dependent pathway is the main mechanism that induces apoptosis post freeze-thawing [64]. Duchez et al. developed a serum-free xeno-free cryoprotectant for cord blood HSPC freezing, which improved cell viability and chimerism in animal models post-thawing [65].

Hornberger et al. reviewed cryopreservation with minimal decreases in viability, including alternative cryoprotectants and technologies to dispose of cryoprotectants prior to infusion [62]. The authors found decreased viability and functionality in cryo-HSPCs and identified that the number of CD34 + cells post-thaw is a good predictor of functionality post infusion. Indeed, the main concern of using thawed HSPCs stems from a decrease in the number of viable cells post-thaw that may limit the effective viable cell dose for hematopoietic reconstitution.

Improvement of cryopreservation techniques and viability may not automatically coincide with high functionality since delayed engraftment can still occur. However, learning from HSCT and improved technologies for quantifying viability and quality of HSPCs with concomitant functional readouts may also help to develop more efficacious cellular therapies [62].

## Mesenchymal Stromal/Stem Cells (MSCs)

MSCs possess profound regenerative and immunomodulatory properties, which are of potential use in the treatment of degenerative, inflammatory, and autoimmune diseases and numerous other pathologies with unmet medical need [2, 12, 66]. MSCs demonstrated an excellent safety profile in early phase clinical trials [2–7, 36, 67, 68]. Regulatory authorities have now approved MSC therapy for perianal fistulas from Crohn’s disease in Europe and steroid-refractory acute Graft versus Host Disease (GvHD) in Japan, and for GvHD in children in Canada and New Zealand [7, 39, 66, 69]. Thus, MSC therapeutics are now well on the way to becoming established therapies in multiple indications [7].

Murata et al. discussed the real-world efficacy of “off-the shelf” MSCs (Temcell^™^) as treatment of refractory acute GvHD [39], a prime indication for MSC therapy [70]. Cryopreserved, “off-the-shelf” MSCs were found to be safe and support a good clinical response (overall response rate of 60%), but additional efforts are still required to increase efficacy and further minimize the cost of cellular therapy. This could be accomplished with optimal cell manufacturing / logistics without compromising quality of cryopreserved cellular products [39]

Despite these advancements of MSC therapy, clinical efficacy is still a major concern [12], since many of the earlier advanced phase clinical trials failed to show clinical benefit [66, 71]. This has created early doubt on the quality attributes of MSC products; especially those thawed from cryopreservation and immediately infused to patients (Figs. 1B and 3) [2, 9•, 12]. Galipeau et al. were among the first to postulate that cryopreservation could be a confounding factor that may explain disparate outcomes between successful preclinical studies and failed clinical trials [17••, 72]. Several studies have demonstrated that freeze-thawing induced cellular injury can be mitigated by alterations in cryopreservation and thawing methodologies [12].

### Freeze-Thawed MSCs Can Exhibit Metabolic Functional Impairment

It has first been demonstrated by Galipeau et al. in the experimental setting as early as 2012, and in 2014 confirmed by Moll et al. in the clinical setting, that thawed MSCs can undergo a substantial heat shock response related to the cryopreservation and freeze-thawing of therapeutic MSC products [9•, 18]. This may lead to a blunted immunomodulatory response to the inflammatory cytokine IFN-γ, which may in turn result in somewhat compromised immunomodulatory properties in clinical trials [17••, 72]. It is of importance to note that the readout in the initial study conducted by Francois et al*.* focused mainly on the role of the key immune regulatory mediator indoleamine 2,3-dioxygenase (IDO) and its responsiveness to stimulation with IFN-γ [18, 73, 74]. This partially reduced immunomodulatory activity of freeze-thawed clinical grade MSC products was in principle confirmed in the clinical follow-up studies by Moll et al. in 2014 [9•, 10], although the overall impairment was found to be modest, and that outcome may also differ between products. Indeed, it is much clearer nowadays that multiple partially redundant/overlapping molecular and cellular mechanisms need to be considered to understand the full picture [66, 75].

### Freeze-Thawed MSCs Can Trigger Stronger Innate and Adaptive Immune Responses

Adjunct to the studies on metabolic impairment, thawed cells were also found to be more susceptible to triggering of the instant blood mediated inflammatory reaction (IBMIR) (Fig. 3) [9•, 10]. The term IBMIR stands for activation of multiple innate immune cascade systems (e.g., complement and coagulation) and subsequent thromboinflammation, which compromises therapeutic cell survival and functionality in vivo, e.g., due to rapid cell loss. It was demonstrated in complement active human serum exposure assays, that thawed MSCs undergo more rapid lysis due to increased complement activation [9•], and it is possible that thawed MSCs undergo more rapid lysis in vivo through IBMR-mediated detrimental processes immediately post-infusion [12].

Moll et al. also compared the in vivo engraftment and clinical response to thawed higher passage MSCs with fresh low-passage MSCs in patients (Fig. 1B) [9•] and they found the latter option to yield a better clinical response rate in patients, while long-term in vivo engraftment was not substantially improved. The hope for improved long-term engraftment was one of the major drivers to conduct this retrospective follow-up analysis. Nonetheless, the short-term migration to inflammatory sites might be substantially impaired for cryostorage-derived versus fresh-from-culture derived MSCs, which are typically employed in animal studies to demonstrate homing to inflammatory sites, as outlined in detailed in the next sections.

In addition, cryopreserved MSCs were shown to undergo apoptosis upon interaction with allogeneic T cells, which suggests that the use of immediately thawed MSCs in allogeneic cell therapy trials may have an add-on negative effect [20]. In turn, Galleu et al. found that triggering of T-cell mediated apoptosis may be a hallmark of MSC-immunomodulation in vivo [31]. Intriguingly, Bashoun et al. found that highest apoptosis and lowest viability peaked at 4 and 24 h post-thawing, respectively, but that there was no difference between fresh and cryo MSCs in functional outcome [76]. Nonetheless, these studies demonstrate that increased T- and NK-cell recognition of thawed vs. fresh MSCs in the clinical setting should be anticipated (Fig. 3).

These observations generally suggest two competing models that have to be weighed against each other depending on the exact disease setting and scenario of therapeutic cell use (e.g., mode of delivery IV infusion vs. IM or other types of tissue injection) [7]: 1) That rapid destruction of MSCs by the hosts’ adaptive and innate system may compromise the efficacy of MSCs infusion in some models and disease indications, and 2) That the induction of apoptosis in infused MSCs and subsequent recognition of the apoptotic cells by host immune cells (e.g., macrophages) may be part of their MoA in vivo [2, 4, 7, 9•, 12, 38, 77, 78].

In the first model, the emphasis would be on the actual need to infuse fairly viable MSCs in the principle sense this therapy has been originally conceived, although long-term engraftment and tissue formation is not given and most likely also not needed [7, 28, 79]. Here, the goal would be to slow down therapeutic cell destruction or to promote therapeutic cell survival. Any benefit in transient therapeutic cell survival would translate in a better therapeutic effect, that would be in principle dependent on the presence of the therapeutic cells for some amount of time (‘hit and run’ mechanism) to initiate a beneficial immune response and healing cascade [7].

In model 1 it would be essential/beneficial that therapeutic cells survive for some time, to induce host immunomodulation / regeneration through cell intrinsic properties specific to MSCs, that cannot easily be substituted with similar efficacy and safety by other cell types. The optimal therapeutic delivery and at least transient presence of living functional MSCs (that are somewhat responsive to the host environment they are transferred to) would be needed for their MoA and efficacy to occur [11, 12]. This could be achieved by multiple means (e.g., cell contact dependent and / or independent mechanism) such as cell surface display and / or secretion of paracrine mediators (e.g., galectin-1 or paracrine growth factors and cellular vesicles, respectively) [7].

Model 1 may be supported by observation derived in the START-1/2 studies conducted by Matthay and coworkers in treatment of ARDS with MSCs [5, 7, 80–82]. Cruz et al*.* reasoned in a later commentary that the failure of the trial may have been related to suboptimal viability (ranging from 36 to 85%) of the cells post thawing for clinical use in treatment of ARDS [80]. A more beneficial response was associated with higher viability of thawed cells before infusion. This would be in line with the original suggestion by Galipeau et al. that cryopreservation and freeze-thawing of MSCs may present an Achilles heel to clinical use of MSC therapeutics in some clinical studies employing systemic infusion of the cells [17••, 72].

Clear simplistic separation of the two models and their relative contribution to therapeutic outcome in different pathology may not always be obvious and require further detailed studies of the in vivo profile of patients treated in clinical studies to define their relative impact [7, 34, 37]. The contribution of apoptotic MSC recognition may be marginal in some pathology, but essential in others. Further proof may be provided by comparing/substituting the MSCs to other similar or distinct cell types (e.g., MSC from different tissue sources vs. fibroblasts vs. ECs vs. PBMCs) or by employing more sophisticated cell modulation/activation/inactivation strategies, e.g., to clarify the individual impact or effect of living, dying, and dead cells [2, 34–37, 83–85].

Akin to recent recognition (model 2) of the involvement of apoptosis and efferocytosis in the antiinflammatory mechanism of MSCs in treatment of acute GvHD [12, 35], also the infusion and silent clearance of apoptotic PBMCs by the host immune system has been explored for many years as antiinflammatory treatment of GvHD and to ameliorate transplant rejection [12, 86, 87]. To emphasize this important point, the exact timing control of the apoptotic vs. necrotic state of the infused cells is essential for a positive outcome when using this approach, since one may be beneficial, but the other potentially harmful to the host/patient [12].

Most importantly, the actual permissiveness of the patient immune system to respond in an appropriate (e.g., antiinflammatory / regenerative) manner to such an apoptotic cell stimulus may be essential and often underestimated [34], e.g., if crucial cellular subsets or subcellular mediators to exert the anticipated beneficial effects are either lacking or unresponsive to the treatment. Here the impact of the host / patient disease course, prior pharmacological and other treatment (e.g., chemotherapy or body irradiation in cancer patients), but also the patients’ immune system education and consequent different immune subset composition in younger vs. older patients (e.g., increasing levels of terminally differentiated T cells (TEMRA)) needs to be anticipated.

### Freeze-Thawed MSCs Can Display Altered Biodistribution and Homing In Vivo

It has been demonstrated that MSCs injected immediately post thawing can show altered biodistribution and homing in vivo (Fig. 3) [19]. These deficits of homing ability of thawed MSCs have been attributed to disruption of the MSCs’ binding to extracellular matrix proteins. Specifically, thawed MSCs demonstrated less ability than fresh MSCs to polymerize F-actin and bind fibronectin and endothelial cells. F-actin is an essential component of the MSCs ability to home to or engraft in the lung, which is the first organ that interacts with the infusion [19].

Similarly, Chabot et al. reported that functionality and viability decreased after thawing and that this correlated with a decrease in cell adhesion molecules [88]. A key difference from other studies is that this was attributed to fluctuating temperature and transient warming events during the cryostorage of frozen cells, rather than the act of cryopreservation and thawing itself (Fig. 2). Transient warming is a common problem in small-scale storage (e.g., Nitrogen tank or freezer), as opposed to the uninterrupted cold-environments with robotic sample retrieval often found in large-scale biobanking (e.g., CB banks). Importantly, Chabot et al. demonstrated that changes in cell surface adhesion molecules could be preserved if transient warming was minimized [89].

In addition, the use of cell coating, hydrogels, and biomaterials may be an effective way to improve the survival of MSCs post thawing. Mao et al. studied a novel approach of mitigating decreased functionality from cryopreservation [40], by employing microgel encapsulation of MSCs prior to cryopreservation. This microgel needs to be sturdy enough to protect the cells from cryopreservation, but should not block secretome diffusion to allow for paracrine interaction and induction of immunomodulation and regeneration. Furthermore, the encapsulation material must withstand the transfusion process and be safe for human use. Mao et al. found in their studies that coating with poly-D-lysine (PDL) fulfilled the different requirements listed above.

Subsequent in vivo studies demonstrated that after infusion of cells into mice, the half-life of encapsulated MSCs in the lung was significantly longer than that of non-encapsulated MSCs. Fresh encapsulated MSCs and cryopreserved encapsulated MSCs were compared and found to have only a slightly different viability and no difference in residence time. In order to mitigate effects of cryopreservation on MSCs’ microenvironment, microgel encapsulation is promising.

Similarly, incorporating the cells in hydrogels may be an effective approach [41]. Drzeniek et al. found that incorporating MSCs into specifically tailored bioinstructive hydrogels with defined pore size expands/optimizes their paracrine potency and this may also improve cell survival post thawing and application in vivo. Thus, hydrogels (coatings) and other types of cell assisting biomaterials can be a beneficial approach for MSC infusion in vivo, which often results in high cell loss due to the harsh in vivo environment the cells encounter upon infusion.

In conclusion, in clinical scenarios where the option of fresh MSCs is not possible, improved cryostorage or use of cell coating / hydrogels for effective cell delivery may be an elegant solution to maximize infusion efficacy to produce a functional “off-the-shelf” product.

### Cellular Senescence Can Impact on the Quality of Cryopreserved MSC Products

Cellular senescence is another confounding factor that may impact on the manufacturing of cryopreserved MSCs. Pollock et al. found that MSC function is detrimentally affected upon thawing from cryopreservation when their pre-freeze counterparts contain higher levels of cellular senescence [90]. This heterogeneity in cryorecovery outcomes depending on the status of cellular senescence appears to be most evident with different batches of BM-MSCs, since BM-MSC products generated and employed in autologous (but less so in allogeneic) biobanking approaches typically show a fairly large heterogeneity in donor material considering donor age and underlying comorbidities, which may also imprint on the resulting MSC products [91–93].

We also observed that clinical batches of placenta-derived decidual stromal cells (DSCs) from a young potent tissue source appear to be more resistant to cryopreservation and subsequent thawing than clinical BM-MSCs batches, that displayed larger heterogeneity, senescence, and more variable functional outcomes in vitro and in vivo [9•, 10, 79, 94]. The clinical DSC batches exhibited improved in vitro growth, post-thaw viability and recovery, and better clinical performance than BM-derived MSCs, which appeared to be more susceptible to freeze-thawing [2, 3, 9•, 10–12, 95–97]. If this improved viability, recovery, and performance is related to the alternative starting material (BM vs. placenta) or the actual manufacturing process needs to be verified in further studies.

### Interpretation of Animal Model Studies on the Impact of Freeze-Thawing on MSCs

Animal model studies to define the potency of fresh and freeze-thawed MSCs need to be interpreted carefully, when considering clinical translation to patients. Preclinical animal model studies often involve infusion of either murine or human donor-derived MSCs in an attempt to test their therapeutic safety and efficacy. Although such studies provide some valuable insights, there are limitations considering their clinical interpretation.

Infusion of murine MSCs does not entirely mimic the clinical setting, since the MoA of human and murine MSCs can be distinct [98]. In addition, murine MSCs may possess unstable genomes, which may skew growth to their immortalization in culture, but this immortalization has not observed with human MSCs [99]. Thus, results from murine MSC studies need to be cautiously transferred into clinical practice, where human MSCs are in use.

Infusion of human MSCs into animals does not entirely mimic the clinical situation either, since xenogenicity is a confounding factor, particularly when studying immunomodulation. It is well established, that MSCs are not fully immune privileged, but “immune evasive” and that they can get rejected by an intact recipient's immune system through MHC-dependent mechanisms [100–102].

It is also often not entirely clear what MoA underlies the therapeutic benefit, which is being observed with human MSCs in animal models. Another major concern is that the human MSCs’ secretome may not entirely interact with the mouse receptors. Hence, it is largely unknown if any therapeutic effect is merely the result of a xenogeneic host response to the infused MSCs or the secretome of infused MSCs.

It has been shown that MSCs infused in animals rapidly undergo apoptosis [31], which may be a crucial part of the MoA contributing to beneficial therapeutic effects in animal models [2, 83–85]. It has been shown that systemic infusion of dead MSCs evokes a host response in animals through a monocyte-dependent phagocytic mechanism [103].

If apoptotic, dying, or dead MSCs are relevant for a therapeutic effect in a given model system and clinical indication, it may be entirely possible that freeze thawing induced apoptosis of MSCs upon infusion might play some beneficial role [2, 12, 37, 83–85].

### Conclusion on Clinical Relevance of Cryopreservation and Freeze-Thawing

There is great clinical and commercial potential for employing cryopreserved MSC products with direct thawing and “bed-side” infusion of MSC products to patients [39, 70], but there are still questions considering optimal manufacturing and standardization of this process. As Bahsoun et al. pointed out [76], cryopreservation of MSCs may be considered an essential part of the process-chain for effective and economically viable distribution to healthcare facilities / patients, but differences in viability and functionality of cryo MSCs versus fresh MSCs cannot be overlooked.

Although some studies support the notion that fresh low-passage MSCs can provide a more efficacious therapy [9•, 10, 12], there are also some preclinical data that propose that thawed MSCs are somewhat equal to fresh MSCs [104–110]. Indeed, the biggest puzzle to solve is the current lack of understanding considering MSCs’ MoA in humans upon infusion [34, 35].

MSCs engage a multitude of immunomodulatory and regenerative pathways [75]. Each of these may be crucial to execute the therapeutic effect depending on the host milieu. More work is needed to better define the in vivo MoA of MSCs’ therapeutic effect in humans. This knowledge will help to define the effect of freeze thawing on MSCs’ in vivo therapeutic effect in humans, and also the optimal manufacturing procedures involving cryopreservation technologies.

## Counteracting Freeze-Thawing Induced Defects on MSCs

**Fig. 4:**
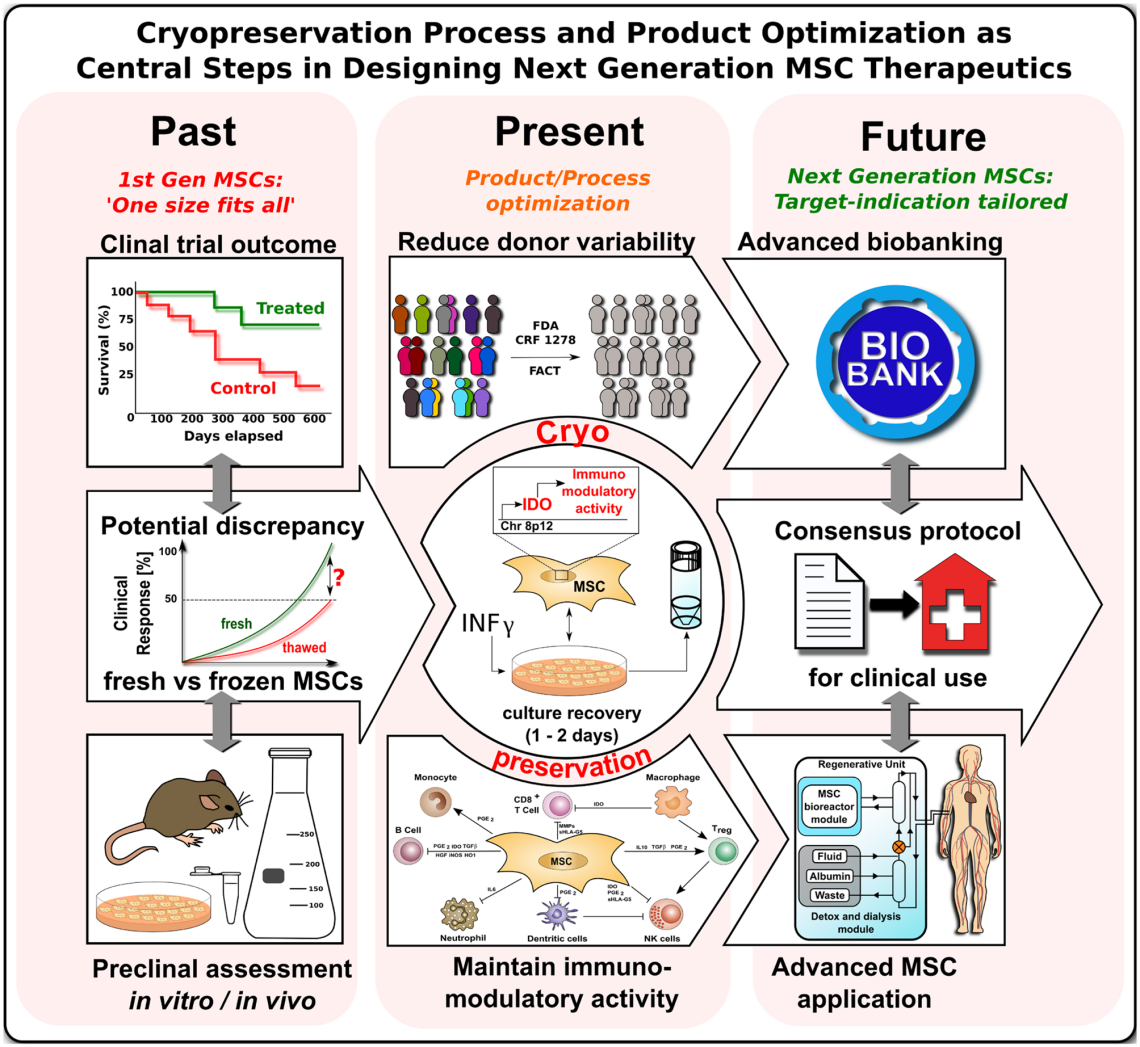
Cryopreservation process and product optimization as central steps in designing next generation MSC therapeutics. A discrepancy in response rates between pre-clinical proof-of-concept studies and clinical trials with mesenchymal stromal / stem cells (MSCs) has been observed in the past [12, 17••, 72]. This discrepancy may be explained by the predominant use of fresh from culture derived MSCs in the pre-clinical proof-of-concept studies as contrasted by the predominant use of cryopreserved MSCs in advanced clinical trials. Major regulatory bodies (e.g., FDA and EMA) demand extensive testing of MSCs for safety and efficacy, thus often making the cryopreservation of MSCs essential to conform to the regulatory standards. The importance of MSC cryopreservation in the past, the present, and the future is evident in the horizontal time line. Four main aspects should be taken into consideration for the future use of MSCs. First, donor variability should be reduced by defined inclusion criteria. Second, cryopreservation should be optimized, e.g., a promising tool to boost immunomodulatory activity is the stimulation of MSCs with IFN-γ prior to initiating the cryopreservation process, which promotes activation / priming of the key immunoregulatory mediator indoleamine 2,3-deoygenase (IDO). Third, culture recovery of cryopreserved MSCs should be performed for 1–2 days prior to in vivo use to restore optimal cellular function. The latter two aspects support the fourth aspect: maintenance of MSC immunomodulatory activity post-transplantation. A new consensus protocol should be established for the clinical use of MSCs, which includes donor specifications, a standardized procedure of the freeze–thaw process of MSCs, requirements for advanced biobanking, and actual considerations on the process of MSC application / delivery in clinical use [3–6, 33, 66]. Due to advances in the development of cardiovascular regenerator (CVR) systems that are compatible with standard dialysis units, cell culture and transplantation may potentially be performed using a single device [33]

There exists a host of literature on the use of fresh-from-culture and cryopreserved freeze-thawed MSC therapeutics and means to improve the outcome accordingly (Figs. 1, 2, 3, 4) [12].

Cryopreserved “off-the-shelf” MSCs are still considered to be the most feasible approach for cellular therapy as a standard of care in clinical practice, mainly due to the logistic and other practical limitations associated with the use of fresh cell products. Nonetheless, it is of importance to investigate effective ways to mitigate or minimize any viability and functionality decrease that freezing and thawing may have on MSCs and other cell types [12].

Not only would this enable larger manufacturing facilities and distribution networks to supply a larger market, but also reduce treatment cost for patients and health-care providers, due to synergy effects resulting from large scale manufacturing and more effective distribution, e.g., centralized cell storage and distribution hubs that share the costly liquid Nitrogen supply.

As suggested earlier, an important strategy to improve viability and engraftment in cell delivery is through cell encapsulation, hydrogels and biomaterial assisted approaches [40, 41]. These play critical roles in current MSC-based engineering approaches, as they allow user control of the biophysical and biochemical extracellular matrix (ECM) signals that can influence MSC behavior and function. In the following sections we will outline key approaches to counteract cryopreservation and freeze-thawing induced defects on MSCs.

### Rate-Controlled Freezing and Thawing Devices and Procedures

Fine-tuning of the cryopreservation process (Fig. 2), in terms of having a set protocol that provides the greatest possible relative amount and total recovery of viable MSCs for cellular therapeutics applications is an obvious first step in mitigating any negative impact related to cryopreservation.

The use of rate-controlled freezing devices / programs / protocols tuned to the particular requirements of specific cell types and freezing vessels (e.g., cryobags or cryovials) has been an important tool in maximizing the viable cell yield available post-thaw. Rate-controlled freezing provides the ability to closely monitor and control the cooling rate, a factor that MSCs and other cell types are very sensitive to, during the cryopreservation process. The precise cooling rates employed by these freezers have reduced variability in the freezing conditions of MSCs and considerably reduced cell injury and death when compared to passive freezing techniques [111].

Controlled-rate-freezing devices are widely recommended to optimize the freezing rate and prevent detrimental osmotic changes of MSCs by controlling the cooling curve enough to prevent ice crystal damage of cells (Fig. 2) [112, 113]. A crucial aspect here is the good fit of the freezing curve / program with the actual volume and dimensions of the respective cryobags and cryovials containing the cells [114]. This requires optimization of the cell concentration in the cryomedium and respective volume requirements and the bag / vial-dimensions to fit the actual cell-dose-demands in the clinic per cryopreserved / thawed cell unit to be employed in patients [12, 114].

Streamlining procedures for optimal thawing of MSCs should also not to be forgotten, e.g., by employing controlled-rate thawing devices. Often, the last step before clinical application, the thawing of cell products at the bedside, is not very well-regulated or cared for and in addition requires properly trained staff. Here it is of importance to maintain optimal cellular viability, recovery, and functionality, with a narrow time window. Some components of the freeze media (e.g., DMSO) can be toxic to the cells once they are thawed, which requires suitable procedures to be in place for fast application. Thus, optimized methodology for both freezing and thawing of MSC products may positively impact the functional recovery of MSCs and their clinical efficacy.

Another crucial aspect is the cryocontainer itself. Wragg et al. studied the reduction of ice nucleation effects on MSCs during freezing by using an Ice Nucleation Device (IND) [115]. The highest cell recovery was accomplished by using the faster thawing rate in combination with the utilization of IND and suitable cryovials, but lower thawing rates in combination with IND produced the best recovery when using micro-well plates. Consequently, this study provided insights not only on the freeze-thawing temperature and the utilization of IND, but also the significance of cryostorage containers.

### Optimized Formulations of Cryoprotectants for Cryomedia

The optimal formulation of the cryoprotectant in which the MSC products are frozen in is a crucial variable in achieving the most efficacious cell therapy product [44, 113, 116–118]. In addition to protecting the MSCs from cellular damage during the cryopreservation process, thought must also be given to actions that must be taken post-thaw to ensure patient safety, and second how these procedures may influence viability and contamination of the final product.

Most cryopreservation approaches for MSCs use a cryomedium containing 10% DMSO as a cytoprotective agent. DMSO is economical and shows great efficacy as a cytoprotective agent, but it can have negative effects towards humans depending on concentration, administration and dose [119]. Grafts containing 30–60% DMSO affect humans with these common side effects including nausea, headaches, hypertension/hypotension, and sedation. Thus, the DMSO amount or concentration used in cryopreservation must either be anticipated by treating physicians when applied to humans (infusion of DMSO containing cells is a rather common procedure), or reduced/removed from solution before infusion for patient safety if doses exceed a toxic threshold [120]. However, this additional step in the procedure increases opportunities for contamination of the product and cell death.

Rogulska et al. set about answering this question by cryopreserving MSCs in a solution of only 1% DMSO in addition to human platelet-poor blood plasma and 0.2 M sucrose [121]. The study showed that this less-toxic and xeno-free solution had a recovery rate that was 73% of non-frozen cells and the recovery rate for the samples cryopreserved in 10% DMSO was 85%. Here, the 12% improved recovery with 10% vs. 1% DMSO needs to be weighed against the anticipated additional cell losses or contamination related to DMSO removal before infusion due to higher toxicity.

Svalgaard et al*.* tested reduction of DMSO concentration by supplementation of alternative cytoprotective additives, such as pentaisomaltose [122]. The results were encouraging, since MSC’s functional recovery post-thaw was improved with DMSO concentration reduced to 1–2% in the presence of additive pentaisomaltose in the cryoprotectant media.

### Culture Rescue as Alternative Use of Fresh MSC Products

Systematic analysis by Oja et al. on the impact of thawing at different stages of MSC production have shown that freezing per se does not compromise MSC products [123]. However, Fresh MSC products can exhibit more potent immunosuppressive effect on T cells than their frozen counterparts, particularly when compared directly post thawing [9•, 18, 20, 76, 123, 124]. This illustrates that freezing per se during MSC production is not the problem, but that methods to regain immunomodulatory function and optimize viability of these products post cryopreservation is an important consideration with regards to achieving maximum therapeutic value of MSCs on a clinically relevant scale [12, 17••].

Oja et al. undertook experiments to explore the effect of a single or multiple freezing steps in the manufacturing process of MSCs on their properties [123], which revealed that a first or second freezing step did not affect the phenotype, proliferation, viability and recovery of MSCs (mean recovery appeared to be reduced upon repeated freezing, but this was not significant), when compared to cells that underwent a single freezing step. Furthermore, repeated freezing steps did not change MSCs’ immunomodulatory properties in vitro, but in agreement with earlier independent studies directly thawed cells performed worse compared to their fresh counterparts before thawing at different stages of culture and passages.

These results by Oja et al. on compromised immunomodulatory activity directly post thaw are in line with earlier independent results by Galipeau et al. and Moll et al. [9•, 18, 20]. Interestingly, several studies also did not observe any compromised viability, recovery, or activity of MSCs directly post thawing in vitro or in vivo in animal models [77, 104, 106–108], which could be interpreted in various ways as discussed in much detail earlier (e.g., “Interpretation of Animal Model Studies on the Impact of Freeze-Thawing on MSCs” section). These studies indicate a potential to improve functional outcomes in the clinical setting when employing improved protocols.

While cryopreservation provides several advantages, fresh MSCs appear to exhibit more consistent/potent immunosuppressive properties than their frozen counterparts. While current cryopreservation techniques allow for sufficient recovery rates of frozen cells to provide some degree of clinical efficacy, a logical next step to potentially achieve more consistent and stronger clinical affects could be to aim for bridging the perceived gap in potency between cryopreserved and fresh cells. Cell culture rescue is a method being investigated for this purpose. Here, the freeze-thawed cells are “recovered or rested” for a certain period post thaw under optimal culture conditions, in order to let them regain their normal state of metabolic activity for optimal clinical efficacy.

Tested time frames for cellular culture recovery typically range between 24 and 72 h. MSC viability and functionality in vitro with or without 24-h cell culture rescue period was analyzed [76, 124]. Both studies confirmed the loss of potency in MSCs directly post thawing associated with cryopreservation, and both studies also demonstrated the efficacy of cell rescue to regain potency following thawing. While potency was completely recovered upon 24-h rescue in the study by Antebi et al. [124], this was not sufficient for MSCs to completely recover in the study by Bahsoun et al. [76], thus indicating a need for further research before a consensus can be reached regarding the duration of maximally effective cell rescue [76, 124].

Another step to increased potency may be to achieve increased survival / retention and biodistribution of the therapeutic cells in vivo, in other words, getting the therapeutic cells to the correct place in the body to exert the desired effects. Chinnadurai et al. studied the effect of culture rescue on biodistribution of MSCs in a mouse model and found that survival and biodistribution of MSCs was significantly increased following a culture rescue period of 48 h when compared to cells that were injected immediately post-thaw [19].

### Pre-freeze Conditioning of MSC Products to Boost Post-thaw Performance

It is possible to increase or boost the immunomodulatory activity of MSCs when exposing them to certain cytokines, a process called conditioning or priming of MSC products [11, 75]. This property has been investigated as a primer in conjunction with cell rescue techniques to increase potency of the final cellular therapeutic product [20, 125, 126]. While it was widely known that exposure to IFN-γ increases the immunomodulatory properties of MSCs [75], Guess et al*.* employed this method as combination of culture rescue and IFN-γ priming post thaw to activate the cells upon recovery from cryopreservation [125]. Importantly, the safety profile of IFN-γ-primed cells was closely comparable to non-primed cells in mouse models.

Another interesting candidate for increasing efficacy is cytokine priming of MSCs prior to cryopreservation, to give them a “jump-start” post thawing (and optional cryorecovery). Specifically, IFN-γ prelicensing was tested on post-thaw functionality of BM-derived MSCs and found to induce persistence permissive chromatin at the IDO promoter [126], thus allowing for more rapid IDO production upon clinical application. Results by Chinnadurai et al*.* demonstrated that IFN-γ prelicensing increases post-thaw MSC survival by inhibiting degranulation of T cells and enhances their immunomodulatory properties to a level comparable with fresh MSCs [20].

A recent study by Mendt et al. on prelicensing of CB-derived MSCs with a cocktail of cytokines provided confirmatory results to the aforementioned IFN-γ prelicensing [20]. Increased immunosuppressive and metabolic activity was observed in the primed group when compared to cells that did not undergo the priming before cryopreservation. When given in mouse models, an increase in survival was observed and GvHD was avoided in the majority of the animals.

We reported on the dichotomic potency of IFN-γ pre-licensed allogeneic MSCs in two different animal models of acute radiation syndrome (ARS) and GvHD [22]. While the application of IFN-γ pre-licensed MSCs protected animals from ARS and radiation-induced lethality by day 30, we did not find a protective effect of IFN-γ pre-licensed MSCs in modulating acute GvHD in a model of major histocompatibility complex (MHC)-mismatched HSCT. This demonstrates that prelicensing may not always overcome a potency defect or refractoriness to MSC treatment in certain model systems or clinical pathologies, respectively.

To boost the efficacy of MSC products and to overcome any risk of compromised cellular unresponsiveness post thawing in patients, the approach of cytokine prelicensing and other similar cellular engineering approaches have now become a crucial component considered in the design of next-generation MSC products employed in clinical trials [127–129].

## Other Common Cellular Therapeutics

As outlined in the previous sections, the research on cryopreservation and freeze-thawing in the field of HSCT, HSPC and MSC therapeutics has been instrumental to improve approaches and outcomes in cellular therapy in general. Much of this knowledge may also be applicable or be transferred to the manufacturing and use of other types of common cellular therapeutics. Here, we will give a brief outline on developments related to cryopreservation and freeze-thawing of products based on T and NK cells and induced pluripotent stem cells (iPSCs).

### T cells and Natural Killer Cells

Cytotoxic T and NK cell-based products are currently employed in > 50% of all clinical cell therapy approaches [130]. Both, T and NK cells are key players of the adaptive and innate immune response, respectively. Preparation of T and NK cell products often requires apheresis and fresh expansion for each application, which makes the process time-consuming, expensive and impractical. Since both T and NK cells are sensitive to cryopreservation, this may also apply to some degree to chimeric antigen receptor (CAR)-modified cell products derived thereof, as studied by Panch et al*.* and summarized by Hanley in their recent publications [29, 30].

Effector T cell (Teff) therapies appear to be more amenable to cryopreservation and freeze-thawing than natural regulatory T cell (nTreg) therapeutics [13, 14•, 15, 16]. While Epstein Barr virus (EBV)-specific Teff’s demonstrated robust viability, recovery, and effector function post thawing, the GMP-manufactured autologous nTreg products demonstrated dismal viability and recovery post thawing (loss of 25–80% of cells within 0–24 h post thaw) when employing the conventional Teff cryopreservation protocol containing 10% DMSO in the cryomedium [14•]. Efforts to optimize cell viability, recovery, and function post thawing demonstrated that reducing the amount of cryoprotectant to 5% DMSO significantly improved the outcome [14•].

NK cells are characterized amongst others by surface expression of CD56 (NCAM), which serves to attach these NK cells to target cells thereby allowing them to exert their cytotoxic effects in a directed manner [131], and in addition, NK cells also produce the proinflammatory effector cytokine IFN-γ [132]. NK cells play a crucial role in the elimination of virally infected, as well as malignant cells as they can respond in a rapid manner to these insults without the need to recognize an MHC molecule in order to be activated [133].

Damodharan et al. studied the cryopreservation of aliquots of freshly expanded NK cells to be thawed and used in subsequent transfusions following the primary fresh NK cell infusion to increase the number of potential applications [133]. Both the viability and the absolute cell number / recovery of the cells were impacted by the freeze-thawing procedure. NKG2D, an important NK cell receptor that plays a key role in NK cell mediated cytotoxicity, was increased in freeze-thawed activated NK cells as well as the freshly activated NK cells before freezing, indicating a certain retention of this function through the cryopreservation process.

Next, the levels of secreted Granzyme B and IFN-γ, which form a key component of NK cell anti-tumor immunity, were examined in fresh and frozen cells, and compared to resting non-activated cells [133]. Elevated levels of Granzyme B were seen in both fresh and frozen activated NK cells, but the levels of IFN-γ did not increase upon activation in the thawed subset.

Surprisingly, IFN-γ even dipped below the basal level observed in non-activated fresh cells. NK cell cytotoxicity was measured against three different cancer cell lines (melanoma, neuroblastoma, and erythroleukemia) [133]. While freeze-thawed NK cells exhibited cytotoxicity against all three cell lines, the fresh activated NK cells were superior, and IFN-γ secretion was reduced in the frozen cell subset.

Mata et al. measured activation and cytotoxicity of NK cells as follows: 1) Fresh cells, 2) frozen/rested cells, and 3) frozen/unrested cells, employing Cr51 and CD107a assays. A steep drop in cytotoxic function was observed from fresh to frozen/unrested NK cells. Interestingly, similar to the prior observations on MSCs, the frozen/rested subset of cells that were allowed to rest/recover for as little as 5 h post thawing, already recovered their cytotoxic functions. This is a principal discovery which could have substantial implications on the way frozen NK cells are handled as a cell therapy to achieve maximum effect [134].

### Induced Pluripotent Stem Cells (iPSCs)

The iPSCs are stem cells that have the ability to be programmed to differentiate into cells of all three germ layers [135–138]. They are of potential use as therapeutics in tissue regeneration (e.g., neural or ocular regeneration) and in disease modeling to study the pathology of diseases at a cellular level [139]. Procedures for cryopreservation of iPSCs usually fall into two categories: slow cooling or vitrification, and further it is of importance to distinguish between freezing of single cells or cell aggregates and the exact mode of freezing and thawing [140, 141].

The slow cooling method entails freezing in a 10% DMSO solution at a low cooling rate, while vitrification uses a high concentration of cryoprotective agent and cooling at a fast rate, to avoid formation of intracellular ice crystals. While slow cooling is technically less challenging, it does not yield the same rates of viable cells post-thaw as vitrification. Refining this procedure may result in greater yields of cells post-thaw, and greater availability of these cells sooner after the cryopreservation process. Li et al. tested parameters for optimal cryopreservation of iPSCs by slow cooling, and found that the lower seeding temperatures (e.g., -7 to -12 °C) commonly seen in the literature are not ideal for the cryopreservation of iPSCs and resulted in a greater amount of intracellular ice formation than seeding at -4 °C [140].

They also found that iPSCs frozen in aggregates were more sensitive to variations in seeding temperature than single cell suspensions. A higher cooling rate (10 °C/minute) resulted in a greater loss of cell membrane integrity than a lower cooling rate (1 or 3 °C/min). Even though higher seeding temperature, lower cooling rate, and cryopreservation of single cell suspensions demonstrated considerable promise when measuring variables such as membrane integrity and intracellular ice formation, apoptosis was still found at a very high rate (over 50%).

Van den Brink et al. used iPSC-derived cardiomyocytes to study the effects of the freeze-thawing procedure [142]. Apoptosis was observed at 24 h after thawing. However, of the cells that did avoid apoptosis and proliferated, the proportion that retained their original characteristics was comparable to that of cells passaged continuously through culture. These results indicate that current procedures can yield functional cells, but that the losses caused during the freeze-thawing procedure could be a logistical challenge when a greater amount of cells are needed in a time sensitive manner and multiplying cells through culture is not an option.

In addition, Li et al. studied cryopreserving iPSCs in an optimized DMSO-free solution compared to the traditional method of using DMSO as cytoprotective agent [143]. The optimized DMSO-free solution reduced the sensitivity of iPSCs to undercooling and allowed more variation in the freeze-thawing protocol without losing cell attachment ability post-thaw, when compared to cryopreserving in a DMSO solution or non-optimized non-DMSO solution.

The cells cryopreserved in the non-DMSO optimized cryosolution retained the ability to differentiate into cells of all three germ layers and had a post-thaw attachment rate comparable to cells passaged in culture (100%). In contrast, the cells cryopreserved with DMSO had a post-thaw reattachment rate of only around 60%. This demonstrates that the use of optimized non-DMSO solutions could be of value to increase the rate at which viable cells can be produced.

In contrast to the more widespread use of the slow cooling method, vitrification is the other major cryopreservation technique used for iPSCs. Vitrification in principle uses a high concentration of cryoprotective agent and cooling at an extremely fast rate, to avoid the formation of intracellular ice crystals altogether. The key issue with vitrification becoming the primary method of cryopreservation for iPSCs is the availability of the technology and the ability to deploy it on a large-scale to allow the widespread clinical use of these cells [140].

Kaindl et al. used the optimized TWIST method to cryopreserve cells and observed greater amounts of confluence and viability of cells after the freeze-thawing process, and the authors concluded that compared to the state-of-the-art slow-rate freezing of single cells in suspension, adherent vitrification is an improved cryopreservation technique for iPSCs and derivatives [144].

The TWIST method uses extremely fast freezing and liquid nitrogen to cryopreserve cells that are still adherent to their TWIST substrate, by employing a device combining cultivation, vitrification, storage, and post-thaw cultivation, all designed in a manner to reduce the potential risk of contamination upon direct contact of the cells with liquid nitrogen.

Importantly, iPSCs have an apoptosis pathway called ROCK that is triggered by a failure of cell attachment and freezing the cells while they are still attached to their substrate alleviates the need to inhibit this pathway pharmacologically. While adding a molecular ROCK inhibitor to the medium of the cells post-thaw can reduce the amount of apoptosis observed in iPSCs post-thaw, it has also been shown to cause chemical and metabolic changes in the cells and cause additional damage. Thus, it is of advantage to freeze the cells adherent to their substrate.

In conclusion, human iPSCs are an important tool for research and regenerative medicine, but their efficient cryopreservation remains a major challenge. Low yield rates of iPSCs from the traditional slow cooling methods have been shown to be detrimental to the quick and efficacious use of iPSCs after cryopreservation, but the technology and resources to perform this method of cryopreservation is more readily available than that of vitrification, and it may be possible to optimize protocols. In contrast, vitrification yields great rates of viable cells post-thaw, but the difficulty of implementing this method on a large-scale will be a big hurdle. While the two methods’ pitfalls are very different, both methods of cryopreservation have obstacles to overcome before they can robustly support the widespread use of iPSCs clinically.

## Conclusion

Both economical and regulatory feasibility are strong arguments for the use of frozen “off-the-shelf” products, if the clinical outcome is satisfactory [39]. Cryopreservation, freeze-thawing, and systemic transplantation of HSPCs are well established and wide-spread procedures. This is in part due to the good amenability and resilience of HSPCs to these challenging procedures. Only a small amount of HSPCs is needed to engraft long-term in their hematopoietic niche in vivo, to perform their regenerative function to reconstitute the hematopoietic system. Their non-hematopoietic MSC counterparts and other cellular therapeutics appear to be more sensitive to freeze-thawing, thus compromising their viability, recovery, and engraftment post cryorecovery. For MSCs only a transient in vivo persistence may be necessary for their effector function to take place upon injection or infusion, e.g., anti-inflammatory/pro-regenerative host response to infusion of apoptotic cells combined with MSC-derived immunomodulatory and regenerative mediators. However, for the progeny of HSPCs long-term engraftment may indeed be desirable, e.g., to protect the host long-term from viral infections with antiviral Teffs, or to elicit anti-cancer or regulatory effector functions by employing CAR-T/-NK or nTreg products, respectively. If cell engraftment is not given, a larger dose of ‘healthy’ cells may be needed for a therapeutic function to take place, as they do not reproduce or increase in number once inside the body. Cellular preactivation (e.g., by using cytokines such as IFN-gamma and other approaches) may yield improved effector function, and optimization of cryopreservation and freeze-thawing methods, to promote optimal cell viability, metabolic activity, and responsiveness, may be crucial for effective clinical application of cellular therapeutics. Further boosting of cellular activity may be achieved by short-term culture-recovery post thawing to reinvigorate their full cellular responsiveness, for the cells to be able to rapidly adapt and respond to the harsh environments they encounter in vivo, before being destroyed by host immune responses (e.g., IBMIR). In conclusion, research into freeze-thawing may provide crucial advantages to increase both the safety and efficacy of cellular therapeutics.
